# Trehalose, sucrose and raffinose are novel activators of autophagy in human keratinocytes through an mTOR-independent pathway

**DOI:** 10.1038/srep28423

**Published:** 2016-06-22

**Authors:** Xu Chen, Min Li, Li Li, Song Xu, Dan Huang, Mei Ju, Ju Huang, Kun Chen, Heng Gu

**Affiliations:** 1 Institute of Dermatology, Jiangsu Key Laboratory of Molecular Biology for Skin Diseases and STIs, Chinese Academy of Medical Science & Peking Union Medical College, Nanjing, 210042, China; 2 Ontario Cancer Institute, University Health Network, Toronto, Ontario, M5G 2M1, Canada

## Abstract

Trehalose is a natural disaccharide that is found in a diverse range of organisms but not in mammals. Autophagy is a process which mediates the sequestration, lysosomal delivery and degradation of proteins and organelles. Studies have shown that trehalose exerts beneficial effects through inducing autophagy in mammalian cells. However, whether trehalose or other saccharides can activate autophagy in keratinocytes is unknown. Here, we found that trehalose treatment increased the LC3-I to LC3-II conversion, acridine orange-stained vacuoles and GFP-LC3B (LC3B protein tagged with green fluorescent protein) puncta in the HaCaT human keratinocyte cell line, indicating autophagy induction. Trehalose-induced autophagy was also observed in primary keratinocytes and the A431 epidermal cancer cell line. mTOR signalling was not affected by trehalose treatment, suggesting that trehalose induced autophagy through an mTOR-independent pathway. mTOR-independent autophagy induction was also observed in HaCaT and HeLa cells treated with sucrose or raffinose but not in glucose, maltose or sorbitol treated HaCaT cells, indicating that autophagy induction was not a general property of saccharides. Finally, although trehalose treatment had an inhibitory effect on cell proliferation, it had a cytoprotective effect on cells exposed to UVB radiation. Our study provides new insight into the saccharide-mediated regulation of autophagy in keratinocytes.

Trehalose is a natural disaccharide that is found in a diverse range of organisms, such as plants, bacteria, yeast and fungi. By contrast, mammalian cells cannot synthesize trehalose. Trehalose has been reported to have a cytoprotective effect on cells subjected to various stressors, including oxidative damage, dehydration and temperature changes^1^. Mitochondria frozen with trehalose have been shown to retain most of their biological properties, such as membrane integrity, ATP synthesis and transmembrane potential^2^. At the molecular level, trehalose has been reported to function as a cryoprotective agent by preventing the crystallization of protein hydration water^3^. Trehalose treatment can protect the cornea against ultraviolet B (UVB) damage by inhibiting the generation of reactive oxygen and nitrogen species^4^. In *Saccharomyces cerevisiae*, trehalose acts as a free-radical scavenger, thereby decreasing the accumulation of abnormal proteins that arises from oxidative stress-induced damage^5^. Additionally, trehalose prevents the amyloidosis of insulin *in vitro*^6^ and represses β-amyloid aggregation in Alzheimer’s disease^7^.

Macroautophagy (hereafter referred to as autophagy) is a cellular degradation process during which double-membrane vesicular structures named autophagosomes are formed and cytosolic components are delivered to the lysosome for breakdown^8^. The autophagy machinery is conserved among eukaryotes from yeast to humans and plays an important role in maintaining cellular homeostasis despite exposure to various types of environmental damage^9^. Abnormal autophagy has been found in many human disorders, including neurodegenerative diseases, metabolic disease, infections and cancer^8^. Accordingly, pharmacological approaches targeted to activate or inhibit autophagy are currently obtaining attention for the treatment of these diseases^10^,^11^. Interestingly, trehalose has been identified as an inducer of autophagy in neural cells^12^,^13^,^14^. Recent studies have shown that trehalose-induced autophagy has many beneficial effects, such as preventing neural tube defects^15^, delaying the progression of amyotrophic lateral sclerosis^13^, ameliorating dopaminergic and tau pathology^16^, counteracting cellular prion infection^14^ and accelerating the clearance of mutant huntingtin and alpha-synuclein^12^.

Mechanistic target of rapamycin (mTOR) is an evolutionarily conserved protein kinase that plays a central role in autophagy regulation and cell growth in response to stress signals, nutritional status and growth factors^17^,^18^,^19^. Unc-51-like kinase 1 (ULK1) is the substrate of mTOR and is activated when mTOR complex 1 (mTORC1) is inhibited under certain conditions, such as nutrient deprivation or rapamycin treatment^20^. Consequently, the active ULK1 protein complex triggers autophagy. mTOR complex 2 (mTORC2) is not directly part of the core autophagy machinery. However, mTORC2 has been speculated to be a negative modulator of autophagy because it directly mediates the phosphorylation and thus the activation of the Akt protein, which is a positive regulator of mTORC1^17^. Autophagy can also be regulated by mTOR-independent pathways^21^,^22^,^23^.

The epidermis of mammalian skin is formed through the successive differentiation of keratinocytes and acts as the front-line barrier against biotic, physical and chemical insults^24^. Recently, autophagy was reported to be involved in keratinocyte cytobiology and pathology, including differentiation^25^, virus infection^26^, melanin metabolism^27^ and the inflammatory response^28^. Sukseree *et al*.^29^ reported that mice that are deficient in keratinocyte-specific autophagy-related gene 5 (ATG5, a key protein associated with autophagy), which have inhibited autolysosomal degradation, presented normal cornification-associated cell death. Moreover, Rossiter *et al*.^30^ found that a significant defect in cornification was not observed in mice that are deficient in keratinocyte-specific ATG7 (a key protein associated with autophagy), which exhibit autophagy suppression in the epidermis, although transepidermal water loss and resistance to penetration of dye were normal in the skin of these mice. These findings indicate that autophagy is not essential for the barrier function of the skin. Interestingly, abnormal presentation of epidermal differentiation was observed in skin grafts of Atg7-deficient mice that had been transplanted onto immunodeficient mice^31^. The epidermis of p18 (a lysosomal adaptor protein)-depleted mice showed severe defects in corneocyte formation and stratum corneum development that were accompanied by the accumulation of autophagosomes and immature lysosomes in the immature corneocyte-like cells. The dysfunction of the fusion between lysosomes and autophagosomes due to p18 ablation led to abnormal development of the mouse epidermis^32^. Therefore, the actual role of autophagy in keratinocytes needs to be clarified. Because the autophagy machinery is a double-edged sword in many disease conditions, it is important to determine the effects of the activation or inhibition of autophagy. Therefore, inducers or inhibitors of autophagy should be used with caution as disease treatment. Recently, some novel inducers of autophagy, including calcipotriol^33^, apigenin^34^, efavirenz^35^ and resveratrol^36^, were identified for keratinocytes. Trehalose has been reported to possess numerous beneficial effects for nervous system diseases via inducing autophagy; however, whether trehalose and other saccharides can activate autophagy in keratinocytes is unknown. Here, we report that treatment with trehalose and other saccharides (sucrose and raffinose) activated autophagy in human keratinocytes and the A431 epidermal cancer cell line through an mTOR-independent pathway. Moreover, trehalose treatment exhibited a cytoprotective effect on cells exposed to UVB radiation, although trehalose treatment itself had an inhibitory effect on cell proliferation.

## Results

### Trehalose treatment activates autophagy and increases the autophagic flux in HaCaT cells

**Figure 1:**
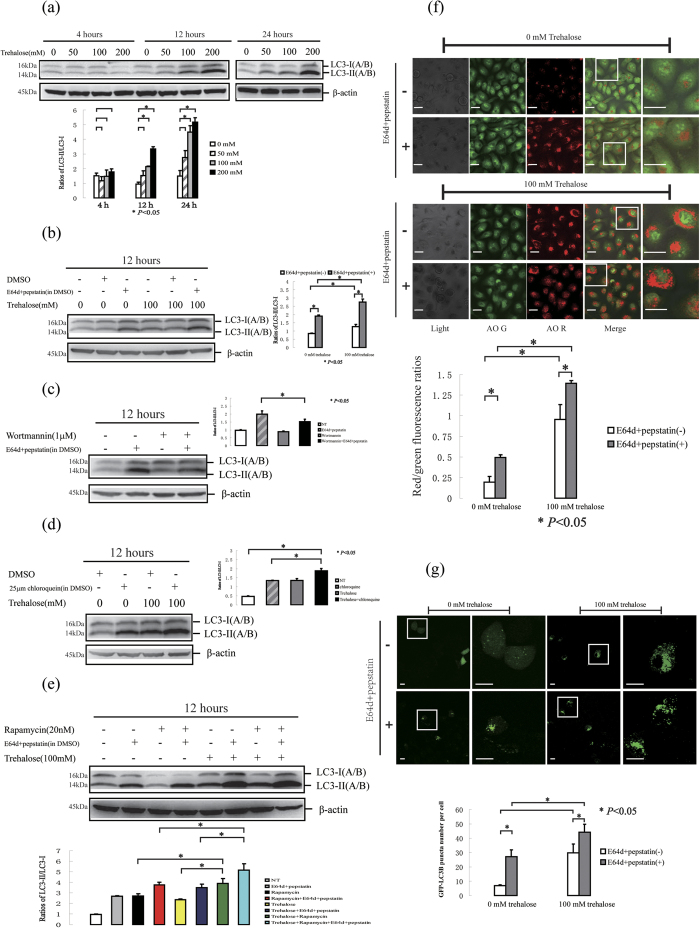
(**a**) HaCaT cells were treated with or without different doses of trehalose for indicated hours. Similar results to (**a**) were repeated in five different passages of cells. (**b**) HaCaT cells were treated with or without 100 mM trehalose for 12 hours in the presence or absence of E64d and pepstatin. 0.01% DMSO (equity for E64d and pepstation solutions) was used as solvent control. Statistical difference of LC3-II/LC3-I ratios was not observed between the DMSO-treated and untreated cells. (**c**) HaCaT cells were treated with or without 1 μM wortmannin for 12 hours in the presence or absence of E64d and pepstatin. (**d**) HaCaT cells were treated with or without 100 mM trehalose for 12 hours in the presence or absence of chloroquine. (**e**) HaCaT cells were treated with or without 100 mM trehalose and rapamycin for 12 hours in the presence or absence of E64d and pepstatin. (**f**) HaCaT cells were treated with or without 100 mM trehalose for 12 hours. Then, cells were incubated with AO. (**g**) HaCaT cells were pretreated with or without GFP-LC3B before trehalose co-treatment for 12 hours. The cells were imaged using a laser scanning confocal microscope, and the means of red/green fluorescence ratios or GFP-LC3 puncta for individual cells were determined for statistical analysis. The data were shown as means ± SD of three independent experiments and representative figures were shown. Bars = 20 μm.

To monitor the autophagy level, we determined the level of microtubule-associated protein 1 light chain 3 (LC3), which is a widely used protein marker of autophagy^17^. The cytosolic form of LC3 (LC3-I) is converted to the phosphatidylethanolamine-conjugated form (LC3-II) when autophagy occurs. In the untreated HaCaT cells (human keratinocyte cell line), we observed basal expression of LC3-I and LC3-II, indicating the basal autophagy level. In a previous study, 100 mM trehalose treatment was reported to increase autophagy in COS-7 cells^12^. Therefore, the cells were treated with 50, 100 and 200 mM trehalose for 4, 12 and 24 hours. We found that the conversion of LC3-I to LC3-II was increased in the cells treated with 100 or 200 mM trehalose for 12 or 24 hours (Fig. 1a).

Cellular autophagy is a highly dynamic multi-step process during which sequestered phagophores, autophagosomes and autolysosomes (formed by the fusion of an autophagosome and lysosome) are sequentially formed during autophagic flux. After degradation in the autolysosome, the autophagy process completes the turnover of the autophagosome and its cargo. The inferred LC3-II turnover is used to measure the autophagic flux, by determining the LC3-II level with or without lysosomal blockade (e.g., in the presence of E64d and pepstatin)^17^. We observed LC3-II accumulation in the presence of E64d and pepstatin for 12 hours, indicating the basal autophagic flux (Fig. 1b). Moreover, the basal autophagic flux can be inhibited by treatment with the autophagy inhibitor wortmannin (Fig. 1c). Increased LC3-II expression was observed after 100 mM trehalose treatment in the presence or absence of E64d and pepstatin (Fig. 1b). Importantly, this effect was more significant in the presence than in the absence of E64d and pepstatin. This effect was verified using the autophagic blocker chloroquine (Fig. 1d). Rapamycin is a widely used inducer of autophagy in mammalian cells. First, we verified that rapamycin treatment increased the autophagic flux in HaCaT cells. Interestingly, the increased conversion of LC3-I to LC3-II was more significant when the cells were simultaneously treated with rapamycin and trehalose than when the cells were treated with rapamycin or trehalose respectively in the presence of E64d and pepstatin, indicating that trehalose had a synergistic effect on the rapamycin-enhanced autophagic flux (Fig. 1e).

Acridine orange (AO) can be used to stain autophagosomes^17^,^37^,^38^. We found that trehalose treatment increased the red/green fluorescence ratios of HaCaT cells in the absence of E64d and pepstatin, indicating an increase in AO-positive vacuole formation. Moreover, the red/green fluorescence ratios were greater in the presence of E64d and pepstatin than in their absence, indicating blockage of autophagosome degradation. Similar to the results obtained from the LC3-II turnover assay, we observed a more significant accumulation of AO-positive vacuoles after trehalose treatment in the presence of protease inhibitors (Fig. 1f).

The GFP-LC3B (the LC3B protein tagged at its N terminus with a green fluorescent protein (GFP)) assay was used to monitor autophagy through fluorescence microscopy by measuring punctate GFP fluorescence^39^. Consistent with the AO staining results, trehalose treatment increased the number of GFP-LC3B puncta in the cells. E64d and pepstatin incubation induced more GFP-LC3B puncta in the trehalose-treated cells than in the cells without lysosomal blockade, indicating the accelerated conversion of LC3-I to LC3-II (Fig. 1g).

These findings strongly suggest that trehalose treatment increases the autophagic flux in HaCaT cells.

### Trehalose treatment induces autophagy of HaCaT cells via an mTOR-independent mechanism

**Figure 2:**
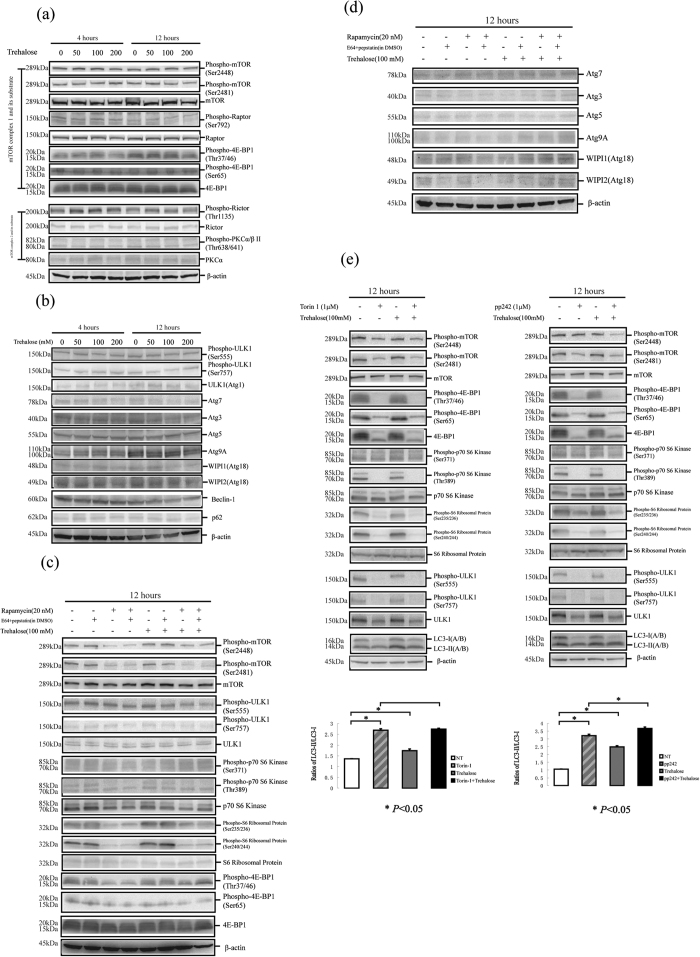
HaCaT cells were treated with or without different doses of trehalose (50, 100 or 200 mM) for 4 or 12 hours. (**a**) Western blotting analysis was performed using primary antibodies against mTOR, phospho-Ser2448 mTOR, phospho-Ser2481 mTOR, Raptor, phospho-Ser792 Raptor, 4E-BP1, phospho-Thr37/46 4E-BP1, phospho-Ser65 4E-BP1, Rictor, phospho-Thr1135 Rictor, Phospho- Thr638/641 PKCα/β or PKCα. (**b**) Western blotting analysis was performed using primary antibodies against ULK1, phospho-Ser555 ULK1, phospho-Ser757 ULK1, ATG7, ATG5, ATG3, ATG9A, WIPI1 or WIPI2. HaCaT cells were treated with or without 100 mM trehalose and rapamycin for 12 hours in the presence or absence of E64d and pepstatin. Then, the activities of mTORC1, ULK1, S6 ribosomal protein phosphorylation (**c**) and ATG proteins (**d**) were determined by western blotting using the indicating primary antibodies. β-actin served as a loading control. Representative figures were shown from three independent experiments.

Trehalose has been reported to be an mTOR-independent autophagic enhancer in cells of the nervous system^12^,^40^. Here, we confirmed that the activities of mTORC1 and mTORC2 were not affected in trehalose-treated HaCaT cells. We found that the phosphorylation levels of mTOR and regulatory-associated protein of mTOR (Raptor, a component of mTORC1) were not affected by trehalose treatment. The phosphorylation levels of the mTORC1 substrate 4E-BP1^41^ at Thr37/46 and Ser65 were also not affected. In addition, trehalose treatment did not affect the phosphorylation of the rapamycin-insensitive companion of mTOR protein (Rictor, a core component of mTORC2^42^) at Thr1135 or of protein kinase C α(PKCα, a substrate of the mTORC2 kinases; mTORC2 facilitates its phosphorylation) at Thr638 (Fig. 2a). Rapamycin-induced autophagy is mediated by an mTOR-dependent mechanism. Therefore, we monitored whether mTOR activity was affected upon trehalose treatment in the presence or absence of rapamycin. In the rapamycin-treated cells, the mTOR phosphorylation levels were decreased and the 4E-BP1, p70 S6 kinase (another important substrate of the mTOR kinase) and S6 ribosomal protein (substrate of the p70 S6 kinase) activities were inhibited, indicating the inhibition of mTOR signalling. However, these results were not observed in the trehalose-treated cells. Interestingly, the inhibition of mTOR activity was observed in cells treated with trehalose and rapamycin together, indicating that trehalose did not affect the rapamycin-induced inhibition of mTOR signalling (Fig. 2c). These findings suggest that trehalose-enhanced autophagy is mediated via an mTOR pathway.

A series of genes reported to be involved in the molecular cellular autophagy machinery have been denoted the autophagy-related genes (ATG). In our study, we determined the unc-51-like kinase 1 (ULK1, yeast ATG1 homologue), ATG3, ATG5, ATG7, ATG9A, Beclin 1, WIPI2 and WIPI1 (yeast ATG18 homologue) protein levels in trehalose-treated HaCaT cells. These proteins were not affected by trehalose treatment. The phosphorylation of ULK1 at Ser555 and Ser757 was also not changed (Fig. 2b,d). These findings indicate that these ATG proteins may not be involved in the autophagic machinery of keratinocytes via regulating the protein levels. In some settings, p62 can be used as a protein marker to monitor autophagy in addition to LC3. However, we did not observe a change in the p62 protein level in the trehalose-treated HaCaT cells (Fig. 2b). Finally, we determined the p62 protein level in human keratinocytes treated with rapamycin or a lysosomal inhibitor (Fig. S1c–e). Our findings verified the conclusion that there was not always a clear correlation between the LC3-II increase and p62 decrease^17^.

Torin 1 and pp242 are mTOR inhibitors^43^,^44^. We found that torin 1 and pp242 both inhibited mTOR activity and subsequently caused an increase in the conversion of LC3-I to LC3-II. Moreover, inhibition of mTOR activity was observed in cells treated with torin 1 or pp242 in the presence or absence of trehalose (Fig. 2e). In agreement with the assay in the cells treated with rapamycin and/or trehalose, these findings demonstrate that mTOR signalling in HaCaT cells was sensitive to the mTOR inhibitors but was not affected by trehalose treatment.

### Trehalose, sucrose and raffinose activate mTOR-independent autophagy in HaCaT cells

**Figure 3:**
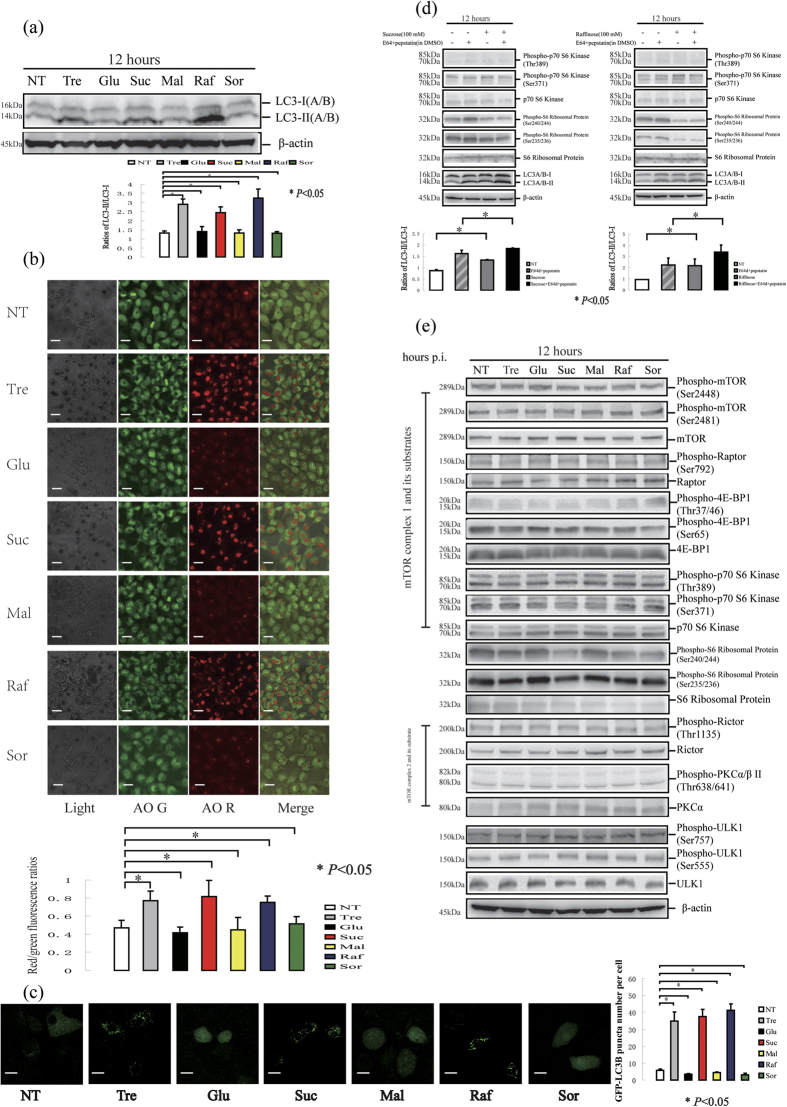
HaCaT cells were treated with or without trehalose (Tre), glucose (Glu), sucrose (Suc), maltose (Mal), raffinose (Raf) or sorbitol (Sor) at the same dose of 100 mM for 12 hours. (**a**) Expression of LC3 protein was determined by western blotting, and then ratios of LC3-II/LC3-I was calculated. β-actin served as a loading control. (**b**) The means of red/green fluorescence ratios for individual cells with or without above saccharides treatment were determined in three independent experiments of AO staining assay. (**c**) HaCaT cells were pretreated with GFP-LC3B before co-treatment of above saccharides for 12 hours. Then, GFP-LC3 puncta per cell was quantified. The cells were imaged using a laser scanning confocal microscope. (**d**) HaCaT cells were treated with or without sucrose or raffinose respectively in the presence or absence of E64d and pepstatin, and the levels of LC3, p70 S6 kinase and S6 ribosomal protein were determined by western blotting. (**e**) HaCaT cells were treated with above saccharides respectively and then the activities of mTORC1, mTORC2, ULK1 and S6 ribosomal protein were determined. β-actin served as a loading control. The data were shown as means ± SD of three independent experiments, and representative figures were shown. Bars = 20 μm.

To investigate whether the trehalose-induced autophagy was caused by osmotic stress, we monitored the autophagy level in HaCaT cells treated with 100 mM of the disaccharide sucrose or maltose for 12 hours. Sucrose treatment increased the conversion of LC3-I to LC3-II, the red/green fluorescence ratios in the AO-stained cells and the GFP-LC3 puncta, indicating autophagy induction. In contrast, a similar effect was not observed in the maltose-treated cells, suggesting that the induction of autophagy was not caused by osmotic stress and was not a general property of disaccharides. Additionally, a 100 mM dose of glucose (monosaccharide) or sorbitol (sugar alcohol) did not induce autophagy. Interestingly, we found that raffinose (trisaccharide) treatment increased the conversion of LC3-I to LC3-II, the red/green fluorescence ratios in the AO-stained cells and the GFP-LC3 puncta, indicating that raffinose induced autophagy (Fig. 3a–c). Moreover, the sucrose or raffinose treatments increased the autophagic flux (Fig. 3d).

The mTORC1, mTORC2 and ULK1 activities were not affected by trehalose, sucrose or raffinose treatment. Interestingly, phosphorylation of the S6 ribosomal protein was decreased in cells treated with sucrose or raffinose, suggesting that these saccharides affected mRNA translation in the HaCaT cells (Fig. 3d,e).

### Trehalose treatment induces autophagy in human primary keratinocytes and the A431 epidermal cancer cell line

**Figure 4:**
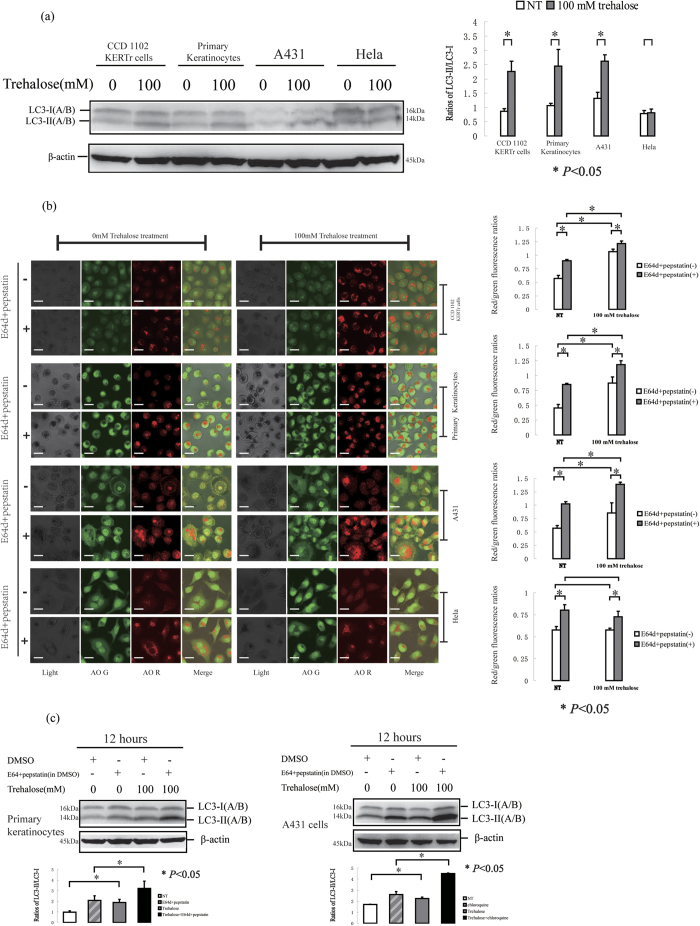
CCD 1102 KERTr cells (keratinocytes transformed HPV 16 E6/E7 oncogenes), human primary keratinocytes, A431 cells and HeLa cells were treated with 100 mM trehalose for 12 hours. (**a**) The expression of LC3 protein was determined by western blotting, and then the ratios of LC3-II/LC3-I was calculated for statistical analysis. β-actin served as a loading control. The cells were imaged for AO staining assay using a laser scanning confocal microscope. (**b**) The means of red/green fluorescence ratios for individual cells in three independent experiments were determined. (**c**) Human primary keratinocytes or A431 cells were treated with or without 100 mM trehalose in the presence or absence of E64d and pepstatin. The levels of LC3 protein were determined by western blotting, and the ratios of LC3-II/LC3-I was calculated. β-actin served as a loading control. The data were shown as means ± SD of three independent experiments, and representative figures were shown. Bars = 20 μm.

**Figure 5:**
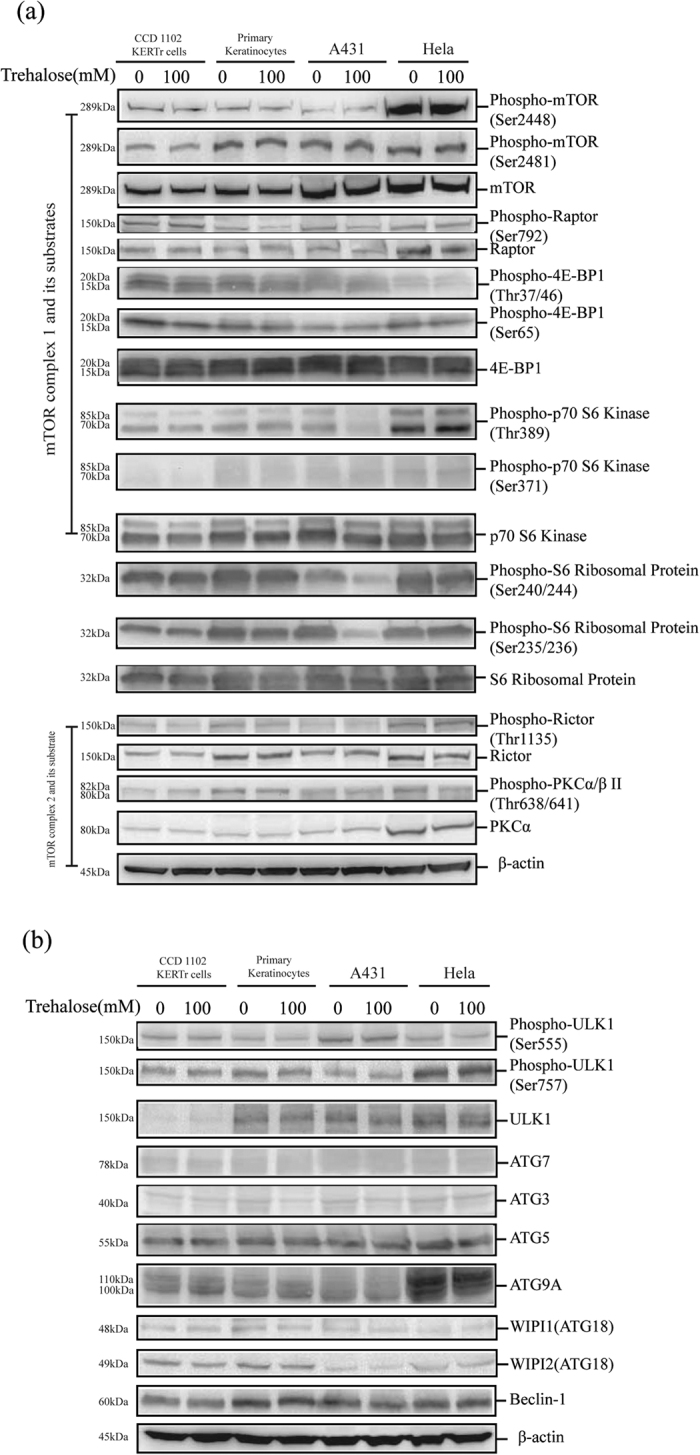
CCD 1102 KERTr cells (keratinocytes transformed HPV 16 E6/E7 oncogenes), human primary keratinocytes, A431 cells and HeLa cells were treated with 100 mM trehalose for 12 hours. Western blotting analysis was performed to determine the activities of mTORC1, mTORC2 and S6 ribosomal protein (**a**) and the levels of ATG proteins (**b**) using indicated primary antibodies. β-actin served as a loading control. Representative figures were shown from three independent experiments.

We monitored the autophagy levels in other human keratinocytes treated with trehalose, including primary keratinocytes, keratinocytes transformed with human papillomavirus 16 (HPV-16) E6/E7 (CCD 1102 KERTr cells), as well as the A431 epidermal cancer cell line^45^ and the HeLa human cervical carcinoma cell line. We found that 100 mM trehalose treatment for 12 hours increased the autophagy levels in these cells with the exception of the HeLa cells (Fig. 4a–d). The mTORC1 and mTORC2 activities were not affected by trehalose treatment in these cells (with the exception of the A431 cells) regardless of whether autophagy was activated. In the trehalose-treated A431 cells, we observed that the phosphorylation of the p70 S6 kinase (an mTOR substrate) and the S6 ribosomal protein was decreased, whereas the phosphorylation of mTORC1 and another substrate (4E-BP1) was not affected. These findings demonstrate the specificity of p70 S6 kinase activity in A431 cells compared with normal keratinocytes. Furthermore, ULK1 activity and some ATG proteins were not affected in the cells treated with trehalose (Fig. 5a,b).

**Figure 6:**
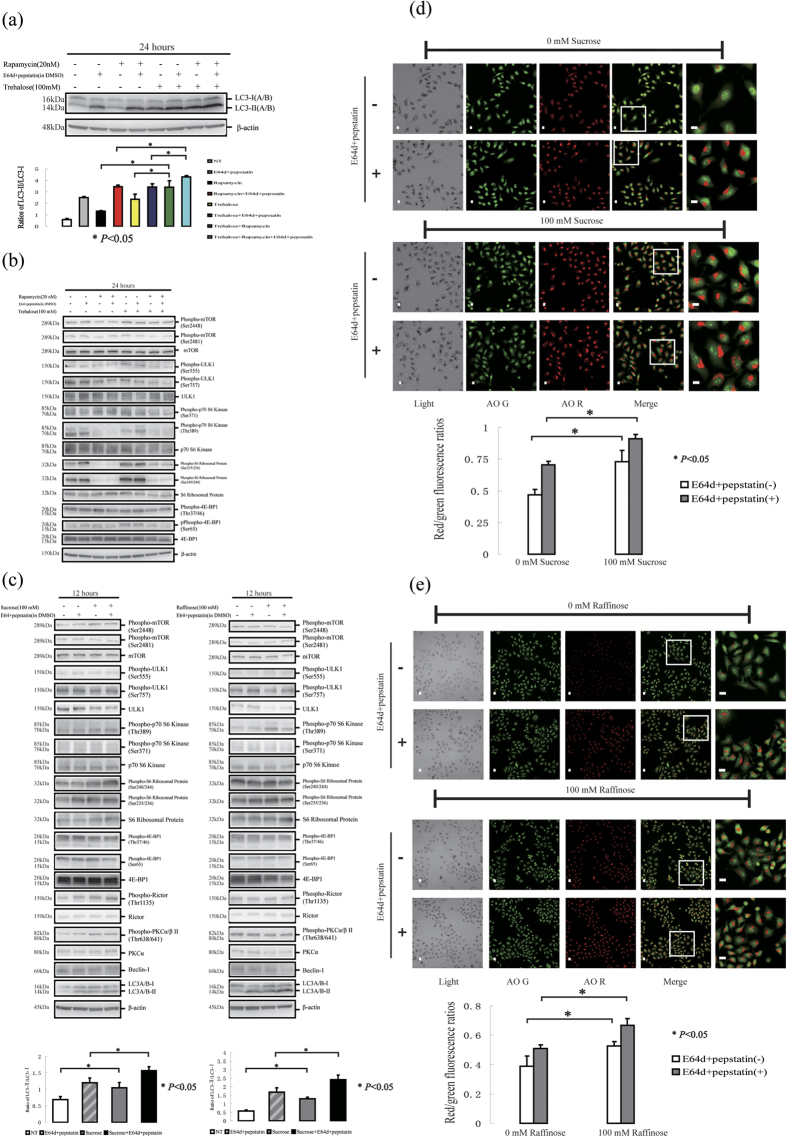
(**a**) HeLa cells were treated with or without 100 mM trehalose and rapamycin for 24 hours in the presence or absence of E64d and pepstatin. The level of LC3 protein was determined by western blotting, and then ratios of LC3-II/LC3-I was calculated. (**b**) Then, activities of mTORC1, ULK1 and S6 ribosomal protein were determined by western blotting. β-actin served as a loading control. HeLa cells were treated with or without sucrose or raffinose in the presence or absence of E64d and pepstatin. (**c**) Western blotting was performed to determine the conversion of LC3-I to LC3-II and activities of mTORC1, mTORC2, ULK1 and S6 ribosomal protein. Additionally, the cells were imaged for AO staining assay using a laser scanning confocal microscope (**d**,**e**). The means of red/green fluorescence ratios for individual cells were determined in three independent experiments of AO staining. The data were shown as means ± SD of three independent experiments, and representative figures were shown. Bars = 20 μm.

Autophagy induction was observed in the HeLa cells when the trehalose incubation time was lengthened to 24 hours, indicating that the HeLa cells were likely to be insensitive to brief treatment with trehalose (Fig. 6a). Indeed, Sarkar *et al*. reported that autophagy was induced in HeLa cells treated with 100 mM trehalose for 24 hours^12^. Moreover, autophagy in the HeLa cells was induced by treatment with sucrose or raffinose (Fig. 6c–e). Importantly, autophagy induction in the HeLa cells treated with trehalose, sucrose or raffinose was mediated via an mTOR-independent way (Fig. 6a–c).

### Trehalose treatment exhibits a cytoprotective effect on cells exposed to UVB radiation

In this study, cell viability was examined using the LDH assay and trypan blue staining, and cell proliferation was detected using the colony formation assay (the methodology was based on references^46^,^47^,^48^). The LDH assay data showed that trehalose treatment for 4, 12, 24 or 48 hours slightly increased LDH release at a stable low level, whereas a 50 mJ/cm^2^ UVB treatment led to an increase in LDH release in a time-dependent manner, with the most significant effect observed 48 hours after exposure. Interestingly, although trehalose treatment only slightly increased LDH release, it significantly attenuated UVB-induced LDH release 48 hours after exposure but had no significant effect 12 to 24 hours after exposure. Furthermore, the trypan blue staining results showed that UVB radiation significantly increased the number of trypan blue-stained cells, whereas this effect was not observed in the trehalose-treated cells. Trehalose treatment for 12 or 24 hours after UVB exposure recovered the increase in the UVB-treated cells stained with trypan blue.

**Figure 7:**
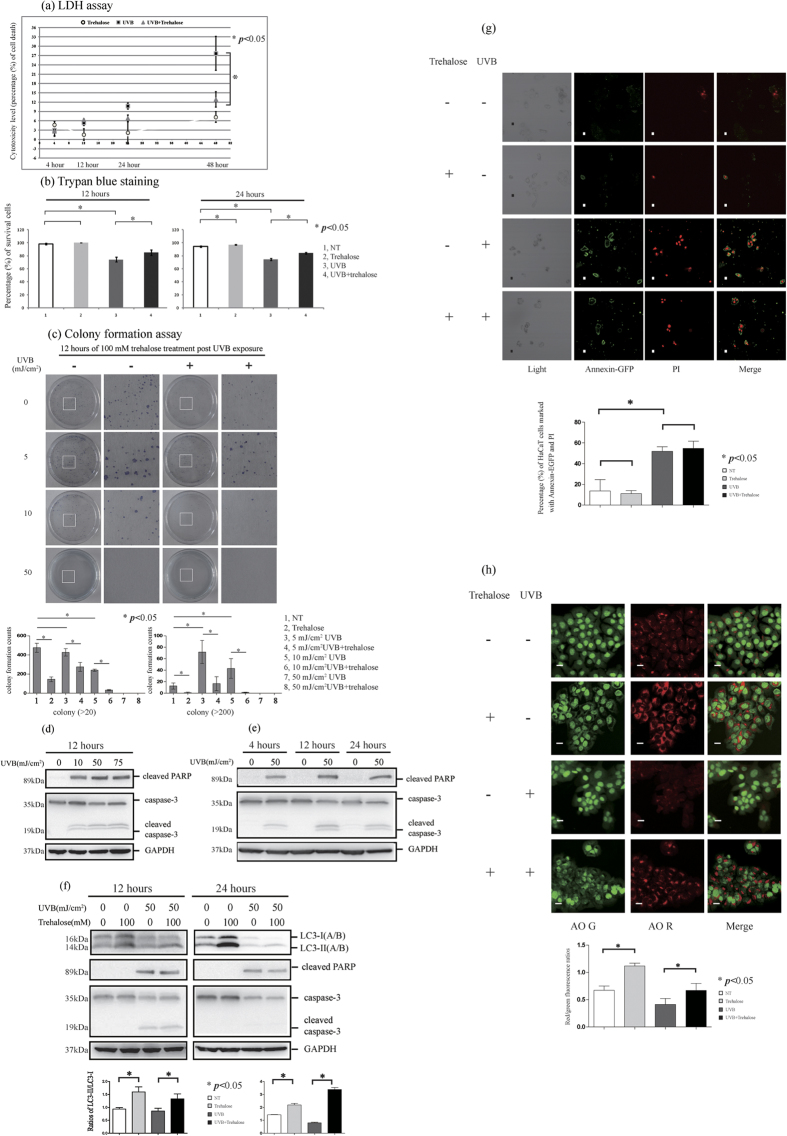
HaCaT cells were treated with or without 50 mJ/cm2 UVB radiation, and then the cells were cultured in the presence or absence of 100 mM trehalose for 4, 12, 24 and 48 hours respectively. Supernatants of the cells culture medium were performed by LDH assay (**A**). Cytotoxicity level values (percentage of cell death) were shown as means ± SD of three independent experiments. HaCaT cells were treated with or without 50 mJ/cm2 UVB radiation, and then the cells were cultured in the presence or absence of 100 mM trehalose. Then, the cells were performed by trypan blue staining (**B**) or by colony formation assay (**C**). The percentages of survival cells (**B**) or colony formation counts (**C**) were shown as means ± SD of three independent experiments. (**D–F**) Western blotting was used to determine the levels of apoptosis molecular markers (cleaved PARP and caspase-3) and LC3 protein. The cells were imaged for AO staining assay or Annexin V-EGFP apoptosis detection using a laser scanning confocal microscope. The percentages of HaCaT cells marked with Annexin-EGFP and PI were calculated (**G**). The means of red/green fluorescence ratios for individual cells were determined (**H**). The means ± SD represented from three independent experiments, and representative figures were shown. Bars = 20 μm.

The LDH assay and trypan blue staining results described above demonstrated that trehalose treatment exhibited a cytoprotective effect on cells exposed to UVB damage (Fig. 7a,b), whereas the colony formation assay results showed that trehalose failed to recover the cell proliferation damage in the UVB-treated cells. First, our data showed that trehalose treatment for 12 hours decreased colony formation, suggesting an inhibitory effect on cell proliferation. Moreover, we did not observe any cell colony formation in the 50 mJ/cm^2^ UVB-treated cells with or without trehalose treatment, indicating that this dose of UVB treatment severely decreased cell proliferation (Fig. 7c).

Interestingly, we found that low doses of UVB (5 or 10 mJ/cm^2^) treatment also decreased ‘normal’ colony formation (>20 cells per colony) and increased ‘excessive’ colony formation, resulting in an increase in the huge colonies (>200 cells). These findings demonstrate that the low dose of UVB stimulation not only impairs normal cell proliferation but also induces ‘undisciplined’ cell proliferation in some surviving cells after UVB exposure. In fact, we detected a low level of ‘excessive’ colony formation in cells under normal culture conditions, and that was decreased by trehalose treatment. Furthermore, trehalose treatment prohibited the increase in ‘excessive’ colony formation caused by 5 or 10 mJ/cm^2^ UVB radiation. These data suggest that the cytoprotective effect of trehalose in the UVB-challenged HaCaT cells reflects the prevention of abnormal excessive proliferation.

Next, we found that 50 mJ/cm^2^ UVB radiation led to more significant cleavage of caspase-3 and PARP than the 10 and 75 dose exposures. Caspase-3 and PARP cleavage was maintained at a stable level after 4, 12 and 24 hours of 50 mJ/cm^2^ UVB exposure; their cleavage levels 12 hours post-radiation were slightly higher than the levels detected at 4 and 24 hours (Fig. 7d,e). In the 50 mJ/cm^2^ UVB-treated cells, the ratio of cells stained with Annexin and PI was also increased 12 hours post-exposure (Fig. 7f,g), suggesting that UVB triggered apoptosis. Interestingly, the increase in cleaved caspase-3, cleaved PARP and cells stained with Annexin and PI 12 hours after 50 mJ/cm^2^ UVB stimulation was not affected by trehalose treatment. We confirmed that the 50 mJ/cm^2^ UVB-induced cleavage of caspase-3 and PARP 24 hours post-exposure was also not affected by trehalose treatment (Fig. 7f,h).

We found that trehalose treatment increased autophagy in HaCaT cells with or without UVB exposure compared with trehalose-untreated cells (Fig. 7f,g).

In conclusion, trehalose treatment had both an inhibitory effect on cell proliferation and a cytoprotective effect on cells exposed to UVB radiation. However, the apoptosis triggered by treatment with 50 mJ/cm^2^ UVB was not affected by trehalose treatment.

## Discussion

Here, we reported that trehalose, sucrose and raffinose were novel mTOR-independent inducers of autophagy in human keratinocytes. Interestingly, this effect is not common to other saccharides.

Autophagy has been strongly implicated in human skin diseases, although limited findings have been reported. First, genetic polymorphisms of some ATG genes have a close relationship with skin diseases. Douroudis *et al*. reported that polymorphisms in the key ATG gene ATG16L1 are associated with psoriasis vulgaris^49^ and palmoplantar pustulosis^50^. Jeong *et al*. reported that polymorphisms of the UVRAG gene, which participates in the autophagy machinery through its interaction with Beclin 1, are associated with susceptibility to non-segmental vitiligo in a Korean sample^51^. Second, autophagy activity may contribute to the epidermal defence against virus infection. Varicella zoster virus (VZV) and herpes simplex virus (HSV) are common pathogens in human skin disorders. Buckingham *et al*. reported that autophagosomes were abundant in transplanted infected skin tissues in a mouse model of VZV pathogenesis. Moreover, autophagy in a VZV-infected cell was significantly distinct from that in an HSV-infected cell^52^. Grose also reported that autophagosomes were observed early in the cells at the base of the cutaneous vesicles caused by VZV infection^53^. Importantly, autophagy has been discovered to play a crucial role in skin cancer, although these reports are extremely rare compared with investigation on tumours of other organs. For example, LC3A-positive ‘stone-like’ structures have been detected in cutaneous squamous cell carcinomas (SCC), indicating that the autophagic activity in SCC may serve as an indicator of tumour aggressiveness^54^. Furthermore, the autophagy inhibitor 3-MA has been shown to enhance sensitivity to 5-FU-induced chemotherapy^55^.

Tong *et al*. reported that apigenin induces keratinocyte autophagy through an mTOR-dependent pathway and is associated with the activation of the AMP-activated protein kinase (AMPK)^34^. Dong *et al*. found that efavirenz triggered keratinocyte autophagy, which was accompanied by a decrease in mTOR phosphorylation and ERK activation^35^. These findings suggest that mTOR plays an important role in autophagy activation in keratinocytes. However, mTOR was not involved in autophagy induction in keratinocytes in response to trehalose, sucrose and raffinose treatment. In contrast with these reported inducers, our findings demonstrate that the autophagy machinery is complex and diverse in human keratinocytes.

Trehalose is a type of disaccharide that consists of two glucose molecules connected through an α, α-1,1-glycosidic linkage. Trehalose is not easily hydrolysed by acid, and glucosidase is not able to cleave its glycosidic bond^1^. However, periplasmic trehalase can catalyse trehalose to hydrolyse trehalose into glucose under high osmolarity conditions; then, the glucose is utilized by the phosphotransferase system for glycolysis in *Escherichia coli*^56^. Otherwise, glucose can also be transformed into trehalose through an acid reversion^57^. If trehalose is not stable in the keratinocyte culture environment and consequently is converted to glucose, autophagy may be induced by the high glucose level. Therefore, we confirmed that 100 mM glucose did not trigger autophagy. The results suggest that the trehalose-induced autophagy may not be caused by the increase in the glucose level generated from trehalose. Interestingly, an increased intracellular glucose concentration has been reported to induce autophagy via an mTOR-dependent pathway in COS-7 cells^58^ or via the ERK pathway in the chick embryo^59^. However, our data indicate that high-level glucose treatment cannot trigger keratinocyte autophagy. Spravchikov *et al*. found that the phosphorylation of insulin-like growth factor receptor decreased when keratinocytes were cultured under high glucose conditions, thereby inhibiting glucose uptake^60^. We speculate that cells derived from different tissues have distinct abilities to uptake external glucose and that human keratinocytes can prohibit the increase in the intracellular glucose concentration under high-level glucose culture conditions. Therefore, the high level of glucose did not trigger keratinocyte autophagy. Importantly, DeBosch *et al*.^61^ reported that trehalose treatment inhibits members of the SLC2A (also known as GLUT) family of the glucose transporters and that trehalose-mediated glucose transport inhibition induces AMPK-dependent autophagy in murine hepatocytes. Therefore, more studies are needed to investigate the signalling pathway associated with glucose transport and metabolism in trehalose-treated cells.

Sucrose and trehalose are both non-reducing disaccharides that possess similarities in synthesis and function. Interestingly, when these oligosaccharides are maintained at a high concentration, cells are more resistant to various stressors, such as oxidative stress, heat and dehydration^62^. Raffinose is an oligosaccharide that can accumulate in some plant cells, which may afford considerable resistance against stresses^62^. Interestingly, Seglen *et al*.^63^,^64^ reported that sucrose and raffinose could accumulate in the cytoplasm and sequester in the autophagosome in mammalian cells. Indeed, radiolabelled sucrose and raffinose are good probes in autophagy studies^63^,^65^. Recently, Higuchi *et al*. reported that sucrose induced the accumulation of sucrose-containing vesicles and autophagy in mouse embryonic fibroblasts^66^. Here, we verified that keratinocyte autophagy was enhanced by sucrose or raffinose treatment. We speculate that keratinocytes promote autophagy to degrade the redundant sucrose or raffinose when these saccharides cannot be utilized after a certain amount of uptake. However, whether trehalose is also the cargo of autophagosomes has not been elucidated.

UVB is radiation at 280–320 nm, in the natural ultraviolet band of the spectrum and is associated with skin conditions such as sunburn and photocarcinogenesis^48^,^67^,^68^. Many studies have reported that various natural agents (e.g., epigallocatechin gallate^69^, pomegranate polyphenol extract^70^, and resveratrol^36^) have photoprotective effects against UV injury. Importantly, Vitale *et al*. found that the anti-photodamage effect of resveratrol was related to the induction of keratinocyte autophagy^36^, suggesting that autophagy regulation was a potential target of photoprotection. Our study indicates that trehalose may be considered as a potential anti-photodamage agent. Importantly, trehalose had an inhibitory effect on the abnormal cell proliferation induced by UVB stimulation. UVB is an important apoptotic trigger for keratinocytes^71^ and the apoptosis mechanism plays a crucial role in prohibiting photocarcinogenesis through the removal of the precancerous cells generated by UV damage^72^. Moreover, there is close interplay between autophagy and apoptosis. For instance, GADD34, which is a protein expressed following DNA damage, suppresses apoptosis in macrophages through enhancement of autophagy^73^. Our findings suggest that the apoptotic molecular markers cleaved caspase-3 and PARP were distinct following different doses of UVB exposure, suggesting that apoptosis could be saturated after a certain dose of UV exposure. Therefore, although we found that the apoptosis triggered by the 50 mJ/cm^2^ UVB dose was not affected by trehalose treatment, the current data did not elicit the conclusion that the trehalose-induced cytoprotective effect observed in UVB-challenged cells was not relevant to apoptosis.

Although Higuchi *et al*. discovered that sucrose-induced autophagy led to the absence of hydrolysis enzymes in mouse embryonic fibroblasts^66^, the mechanism by which trehalose, sucrose and raffinose activate autophagy is unclear. Interestingly, Martinez-Lopez *et al*. reported that ERK phosphorylation was increased by acute trehalose exposure for 2 hours, although prolonged exposure (12 or 24 hours) failed to increase ERK phosphorylation^74^. Their results indicated that trehalose-induced ERK phosphorylation was dependent on autophagy because ERK phosphorylation was not observed in ATG5^−/−^ mouse embryonic fibroblasts. Moreover, Wei *et al*. found that abrogation of ERK phosphorylation by U0126 did not affect trehalose-induced autophagy^75^. Therefore, ERK, which is closely related to autophagy regulation, does not mediate trehalose-induced autophagy. In a recent promising report by Tanji *et al*., trehalose treatment increased the expression of several chaperone molecules, including HSP90 and SigmaR1, and Beclin 1 phosphorylation in mice, although the authors did not confirm whether these effects led to autophagy induction^76^. Additionally, He *et al*. reported that trehalose inhibits NF-κB activation by LPS-mediated stimulation^77^, and NF-kB activation has been reported to be involved in autophagy regulation^78^. Taken together, these results suggest that the roles of the chaperone molecules, the NF-kB pathway and the Beclin 1 complex in trehalose-activated autophagy warrant attention in future studies.

Here, our work verified that trehalose, sucrose and raffinose do not affect the activity of mTOR, which is a key regulator of cell metabolism, proliferation and survival, even though autophagy was activated. Indeed, clinical application of mTOR-dependent autophagy inducers, such as rapamycin, must take into account their effect on mTOR inhibition because cellular functions besides autophagy are potentially disturbed^79^. For example, Chaoul N *et al*.^80^ reported that rapamycin affects cancer vaccine therapy by impairing antitumour CD8+ T cell responses. Therefore, our findings about trehalose, sucrose and raffinose and their mTOR-independent regulation of autophagy have notable implications for human disease therapy.

## Materials and Methods

### Cell culture

HaCaT cells (described previously^81^), A431 cells and HeLa cells (from the Cell Resource Centre, IBMS, Chinese Academy of Medical Sciences and Peking Union Medical College) were cultured in Dulbecco’s modified Eagle’s medium (DMEM) supplemented with foetal bovine serum (Gibco, Invitrogen Corp., Carlsbad, CA, USA). Primary human keratinocytes (described previously^82^) and CCD 1102 KERTr cells (ATCC Number CRL-2310) were cultured in Keratinocyte-SFM Medium (Gibco, Invitrogen Corp., Carlsbad, CA, USA).

### Reagents and antibodies

The compounds used in this study included trehalose (T0167), glucose (G8270), sucrose (84097), maltose (M5885), raffinose (R0250), sorbitol (240850), 10 μg/mL of E64d, 10 μg/mL of pepstatin, rapamycin, chloroquine, dimethyl sulfoxide, acridine orange (AO, A8097), and thiazolyl blue tetrazolium (M5655) (all from Sigma-Aldrich, St. Louis, MO, USA). Other compounds included wortmannin (Santa Cruz, Dallas, TX, USA), Torin 1 (Tocris, Bristol, UK) and pp242 (abcam, Cambridge, MA, USA). Primary antibodies included anti-LC3A/B (#4108), anti-β-Actin (#8457), anti-mTOR (#2983), anti-phospho-mTOR Ser2481 (#2974), anti-phospho-mTOR Ser2448 (#5536), anti-Raptor (#2280), anti-phospho-Raptor Ser792 (#2083), anti-4E-BP1 (#9644), anti-phospho-4E-BP1 Thr37/46 (#2855), anti-phospho-4E-BP1 Ser65 (#9456), anti-Rictor (#2114), anti-phospho-Rictor Thr1135 (#3806), anti-PKCα (#2056), anti-phospho-PKCα/βII Thr638/641 (#9375), anti-phospho-ULK1 Ser555 (#5869), anti-phospho-ULK1 Ser757 (#6888), anti-ULK1 (#4773), anti-Atg7 (#8558), anti-Atg3 (#3415), anti-Atg5 (#8540), anti-Atg9A (#13509), anti-WIPI1 (#12124), anti-WIPI2 (#8567), anti-phospho-p70 S6 kinase Thr389 (#9234), anti-phospho-p70 S6 kinase Ser371 (#9208), anti-p70 S6 kinase (#2708), anti-S6 ribosomal protein (#2217), anti-phospho-S6 ribosomal protein Ser240/244 (#5364), anti-phospho-S6 ribosomal protein Ser235/236 (#4858), anti-cleaved PARP Asp214 (#5625) and anti-Caspase-3 (#9665) (all from Cell Signaling Technology, Danvers, MA, USA).

### Western blotting

The cells were lysed in RIPA Lysis Buffer containing Protease Inhibitor Cocktail and the phosphatase inhibitor PhosSTOP (both from Roche Applied Science, Basel, Switzerland). The protein concentration of the whole cell lysate was determined by the BCA assay (Beyotime Biotechnology, Haimen, Jiangsu, China). Proteins from the cell lysates were loaded onto 4–15% Mini-PROTEAN TGX precast polyacrylamide gels (Bio-Rad Laboratories, Hercules, CA, USA). Then, the proteins were transferred onto PVDF membranes (Bio-Rad Laboratories). After blocking, the membranes were sequentially incubated with the indicated primary and secondary antibodies (Cell Signaling Technology). The protein bands were visualized using an Immun-Star WesternC Chemiluminescence Kit (170–5070, Bio-Rad Laboratories) using a chemiluminescence imaging method. The band intensities were quantified using Quantity One. β-Actin or GAPDH severed as the loading control. LC3 Control Cell Extracts (#11972) and p70 S6 Kinase Control Cell Extracts (#9203) (Cell Signaling Technology) were used to identify the sensitivity of antibodies, including anti-LC3, anti-phosphorylated mTOR and anti-substrates of mTOR (Fig. S1a,b).

### GFP-LC3B puncta analysis

A transgene for GFP-LC3B or GFP-LC3 (G120A) was transfected into cells using the Premo Autophagy Sensor LC3B-GFP BacMam 2.0 system according to the manufacturer’s instructions (P36235, Invitrogen Corp., Carlsbad, CA, USA). Chloroquine was used to evaluate the sensitivity of human keratinocytes to this system (Fig. S2a). LC3B (G120A)-GFP served as the negative control. Before detection, the cells were transfected for at least 24 hours. Next, cells with or without treatment were imaged using an OLYMPUS FV1000 laser scanning confocal microscope as follows: GFP scanning, λ_ex_ = 530 nm and λ_em_ = 500 nm. In all cases, the number of GFP-LC3B puncta per cell was counted using the ImageJ software ( http://imagej.nih.gov/ij/).

### AO staining assay

The AO staining assay was employed to monitor the autophagy process by labelling acidic vesicular organelles (AVO) based on the theory that autophagosomes are vacuole structures that belong to the AVOs. In the AO-stained cells, the nuclei and cytoplasm showed deep green and slight green fluorescence, respectively, whereas the AVOs were marked with red fluorescence. Thus, the intensity of the red fluorescence was proportional to the AVO volume. Comparison of the mean red/green fluorescence ratios per cell between different populations was used to monitor the autophagy level. After treatment, the cells were washed with PBS and incubated with AO (5 μg/mL for 10 minutes) at room temperature. The cells were imaged using a laser scanning confocal microscope (AO G: λ_ex_ = 488 nm and λ_em_ = 515 nm; AO R: λ_ex_ = 546 nm and λ_em_ = 620 nm). The red and green fluorescence intensities in the cells were determined by Quantity One. The red/green fluorescence ratios for individual cells in three independent experiments were used to determine the mean and the statistical significance of the differences was analysed^17^,^37^,^38^. Rapamycin and wortmannin treatment and Earle’s balanced salt solution (EBSS, used for nutrient starvation) incubation were used to confirm the sensitivity of this assay for monitoring autophagy regulation in human keratinocytes (Fig. S2B).

### Lactate dehydrogenase (LDH) assay

The LDH assay was employed to evaluate cell death based on the idea that LDH would be released from the cytoplasm through the damaged plasma membrane when cell death occurred^46^. As a result, quantification of the LDH level in cell culture supernatants was used to evaluate cell death in our study with an LDH cytotoxicity assay detection kit (Beyotime Biotechnology) according to the manufacturer’s instructions. Briefly, HaCaT cells were treated with or without 50 mJ/cm^2^ UVB radiation; then, the cells were cultured in the presence or absence of 100 mM trehalose for 12 hours. Cell culture supernatants were collected for measurement. The optical density was measured at a 490 nm wavelength. The percentage of cell death was calculated with the formula: Cytotoxicity level (percentage of cell death) = (OD of experimental release (trehalose treatment, UVB exposure, or trehalose treatment after UVB exposure, respectively) - OD of spontaneous release (cell with normal culture))/(OD of maximum release (cells treated with LDH releasing reagent provided in kit) - OD of spontaneous release (cell with normal culture)).

### Trypan blue staining

HaCaT cells were treated with or without 50 mJ/cm^2^ UVB radiation; then, the cells were cultured in the presence or absence of 100 mM trehalose for 12 or 24 hours. Subsequently, the cells were digested and stained using a Trypan blue staining cell viability assay kit (Beyotime Biotechnology) to assess cell viability in a blood cell counting chamber according to the manufacturer’s instructions^47^. The percentage of surviving cells = (total number of cells – number of trypan blue positive cells)/total number of cells.

### Colony formation assay

HaCaT cells were treated with or without 50 mJ/cm^2^ UVB radiation; then, the cells were cultured in the presence or absence of 100 mM trehalose for 12 hours. Subsequently, the different groups of cells were seeded into 6 cm tissue culture dishes with 800 cells per dish in 6 ml of DMEM. After culture for 10 days at 37 °C, the cells were washed with warm PBS and fixed in 100% methanol for 5 minutes. Then, the cells were Giemsa stained for 10 minutes to visualize the cell colonies. A colony was defined as a cluster of more than 20 cells. The images were acquired using a Canon EOS 100D digital camera. We conducted the colony formation assay in triplicate to assess the statistical significance.

### UVB radiation

UV light between 290 and 320 nm with a peak at 310 nm was delivered by a UVB irradiation apparatus with a UVB lamp (Philips UVB Broadband PL-S 9W/12). The irradiance was 1.50 mW/cm^2^ at a distance of 16 cm. The cells were irradiated for 6.7, 33.3 or 50 seconds at the 10, 50 or 75 mJ/cm^2^ exposure doses. After irradiation, the cells were incubated with fresh medium with or without trehalose.

### Annexin V-EGFP Apoptosis Detection

Apoptotic HaCaT cells were identified using an Annexin V-EGFP Apoptosis Detection Kit (Beyotime Biotechnology). After treatment, the cells were incubated with Annexin-EGFP and propidium iodide (PI). The membranes of the apoptotic cells were stained with Annexin-EGFP. The nuclei of non-viable apoptotic cells were stained with PI accompanied by the Annexin-EGFP staining. The cells were imaged using a laser scanning confocal microscope. The percentage of apoptotic cells was calculated from three independent experiments and the statistical significance of the differences was analysed.

### Statistical Analysis

The independent experiments were performed at different times. Similar results were obtained from at least three independent experiments for the statistical analysis. The data were analysed by univariate ANOVA or Student’s t-test. Statistical significance was identified as a *P* value of less than 0.05.

## Additional Information

**How to cite this article**: Chen, X. *et al*. Trehalose, sucrose and raffinose are novel activators of autophagy in human keratinocytes through an mTOR-independent pathway. *Sci. Rep.* **6**, 28423; doi: 10.1038/srep28423 (2016).

## Supplementary Material

Supplementary Information
