# Supplementary material for: Trehalose, sucrose and raffinose are novel activators of autophagy in human keratinocytes through an mTOR-independent pathway
Source: Sci Rep. 2016 Jun 22;6:28423. doi: 10.1038/srep28423 (PMC4916512; doi:10.1038/srep28423)

Title page

Trehalose, sucrose and raffinose are novel activators of autophagy in human keratinocytes through an mTOR-independent pathway

Xu Chen[1*], Min Li[1*], Li Li[1], Song Xu[1], Dan Huang[1], Mei Ju[1], Ju Huang[2], Kun Chen[1#], Heng Gu[1#]

1. Institute of Dermatology, Jiangsu Key Laboratory of Molecular Biology for Skin Diseases and STIs, Chinese Academy of Medical Science & Peking Union Medical College, Nanjing, 210042, China

2. Ontario Cancer Institute, University Health Network, Toronto, Ontario, M5G 2M1, Canada

#Corresponding authors:

Heng Gu doctor_guheng@hotmail.com Tel: +86 25 8547 8012

Kun Chen kunchen181@aliyun.com Tel: +86 25 8547 8969

Jiangsu Key Laboratory of Molecular Biology for Skin Diseases and STIs, Institute of Dermatology, Chinese Academy of Medical Science & Peking Union Medical College, 12 Jiangwangmiao St., Nanjing 210042, China

Fax: +86 25 85414477

*: These authors contributed equally to this study.

Supplemental Information

Figure.S1 legend

(**A**) Total cell extracts from HeLa cells untreated or treated with 50 μm chloroquine was subjected to western blot, together with two cell lysate samples from human primary keratinocytes with normal culture. LC3 protein level was determined and GAPDH served as a loading control. (**B**) Total cell extracts from MCF-7 cells untreated or treated with insulin. The protein levels of mTOR, p70 S6 Kinase, S6 Ribosomal protein and 4E-BP1, as well as their phosphorylation levels were detected. This experiment was performed to identify the sensitivity of primary antibodies. β-actin served as a loading control. HaCaT cells (**C**) or primary keratinocytes (**D**) treated with or without 20 or 80 nm rapamycin. (**E**) Primary keratinocytes was treated with or without E64d and pepstatin. Then, the protein levels of LC3 and p62 were determined. GAPDH served as a loading control. The cells treated with DMSO served as a vehicle control.

Figure.S2 legend

(**A**) The transgenes of GFP-LC3B or GFP-LC3 (G120A) were transfected into primary keratinocytes through the Premo Autophagy Sensor LC3B-GFP BacMam 2.0 system according to the instructions of manufacturer. Then the cells transfected with GFP-LC3B or GFP-LC3 (G120A) were treated with 50 μm chloroquine. LC3B (G120A)-GFP served as a negative control. The cells were scanned to monitor GFP-LC3B puncta using a laser scanning confocal microscope. (**B**) Primary keratinocytes were treated with or without rapamycin or EBSS in the presence or absence of wortmannin. After treatment, the cells were incubated with AO, and then scanned using a laser scanning confocal microscope. In the AO G pictures, nucleus and cytoplasm showed deep green and slight green fluorescence respectively. In the AO R pictures, acidic vesicular organelles were marked with red fluorescence. Bars = 20 μm.

Figure.S3 legend

Primary keratinocytes were treated with or without rapamycin (an autophagy inducer) or Earle's balanced salt solution (EBSS, starvation-induced autophagy) in the presence or absence of wortmannin (an autophagy inhibitor). The protein levels of LC3 and Beclin-1 were determined, and GAPDH served as a loading control. We did not observe change of Beclin-1 expression in human primary keratinocytes after treatment of rapamycin or EBSS incubation in the presence or absence of wortmannin, while autophagy can be activated or inhibited. These findings indicated that Beclin-1 was not involved in canonical autophagy regulation of keratinocytes through modifying its protein expression.

Figure S1

Figure S1a

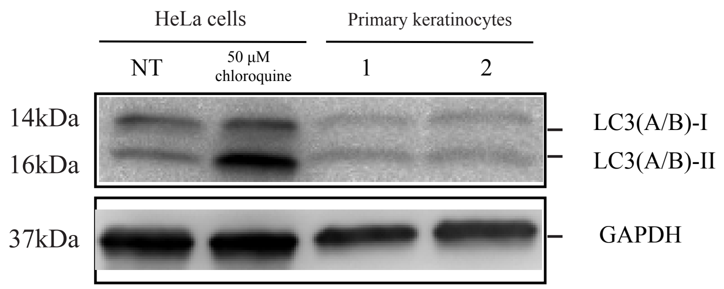

Figure S1b

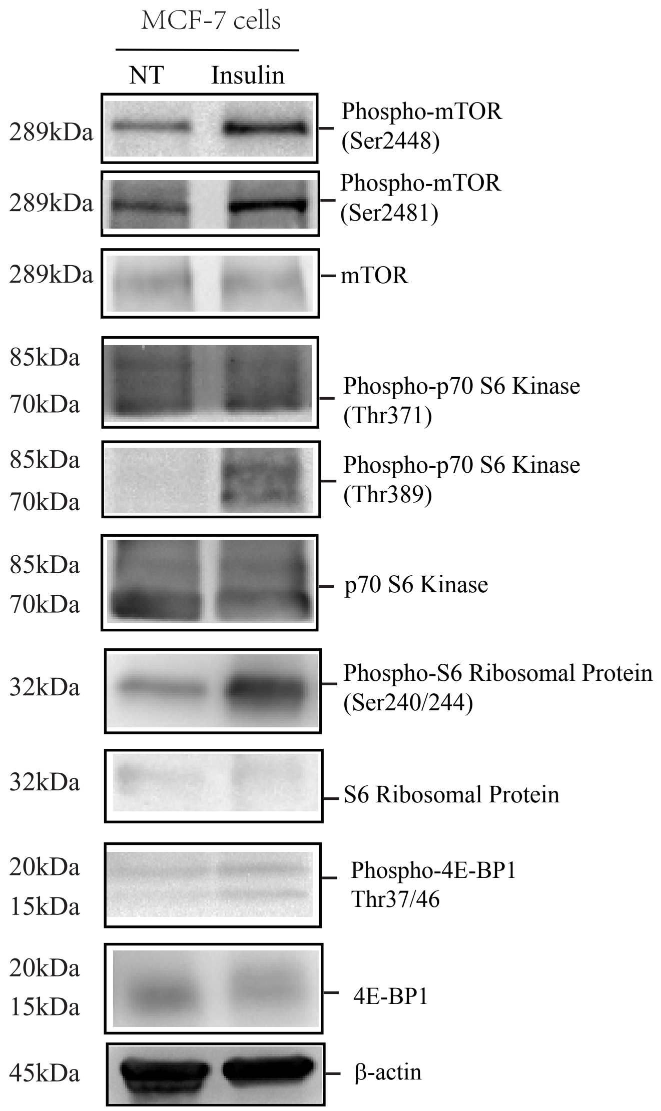

Figure S1c

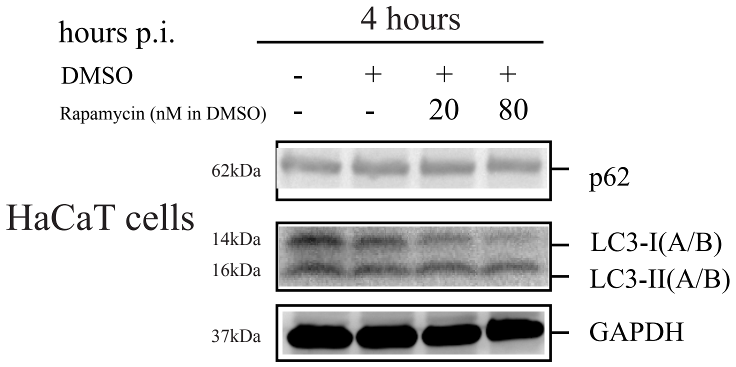

Figure S1d

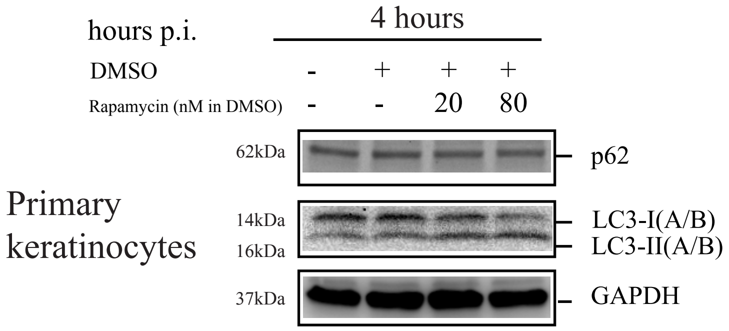

Figure S1e

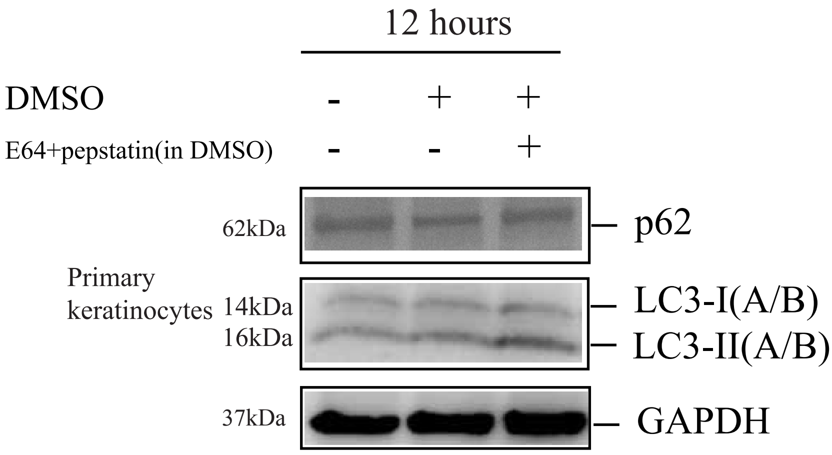

Figure S2

Figure S2a

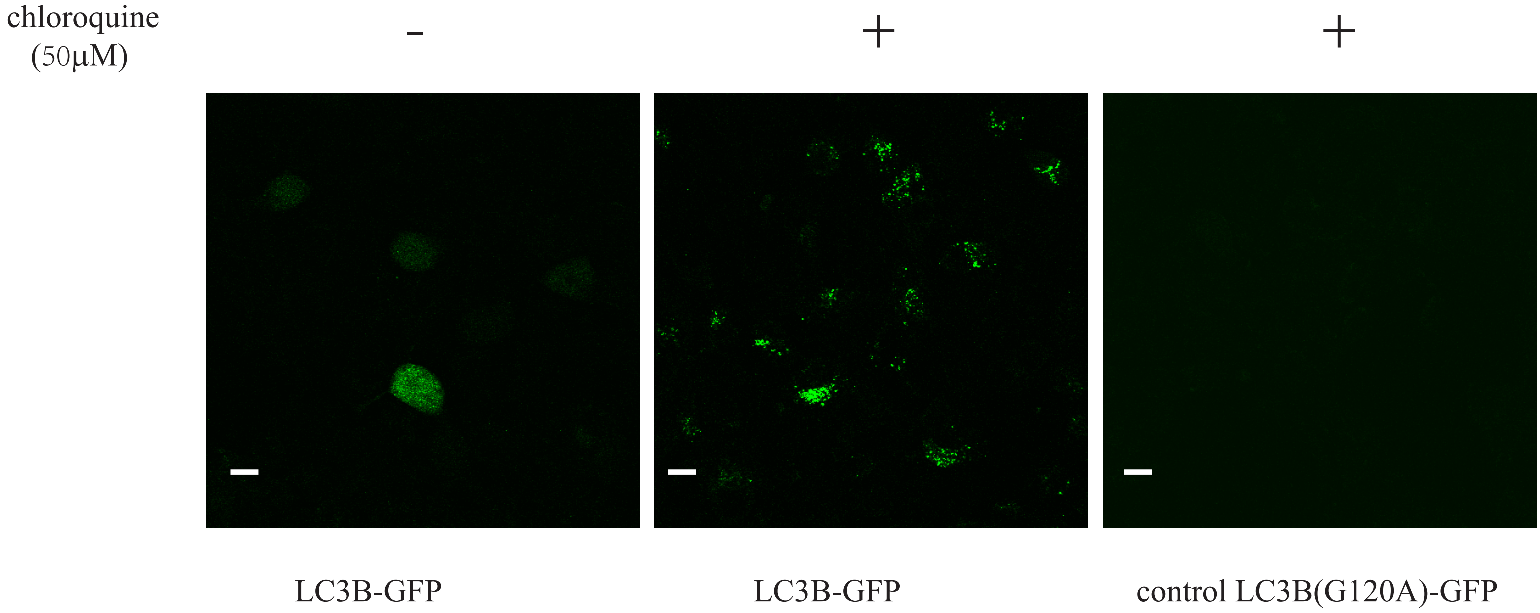

Bars=20μm

Figure S2b

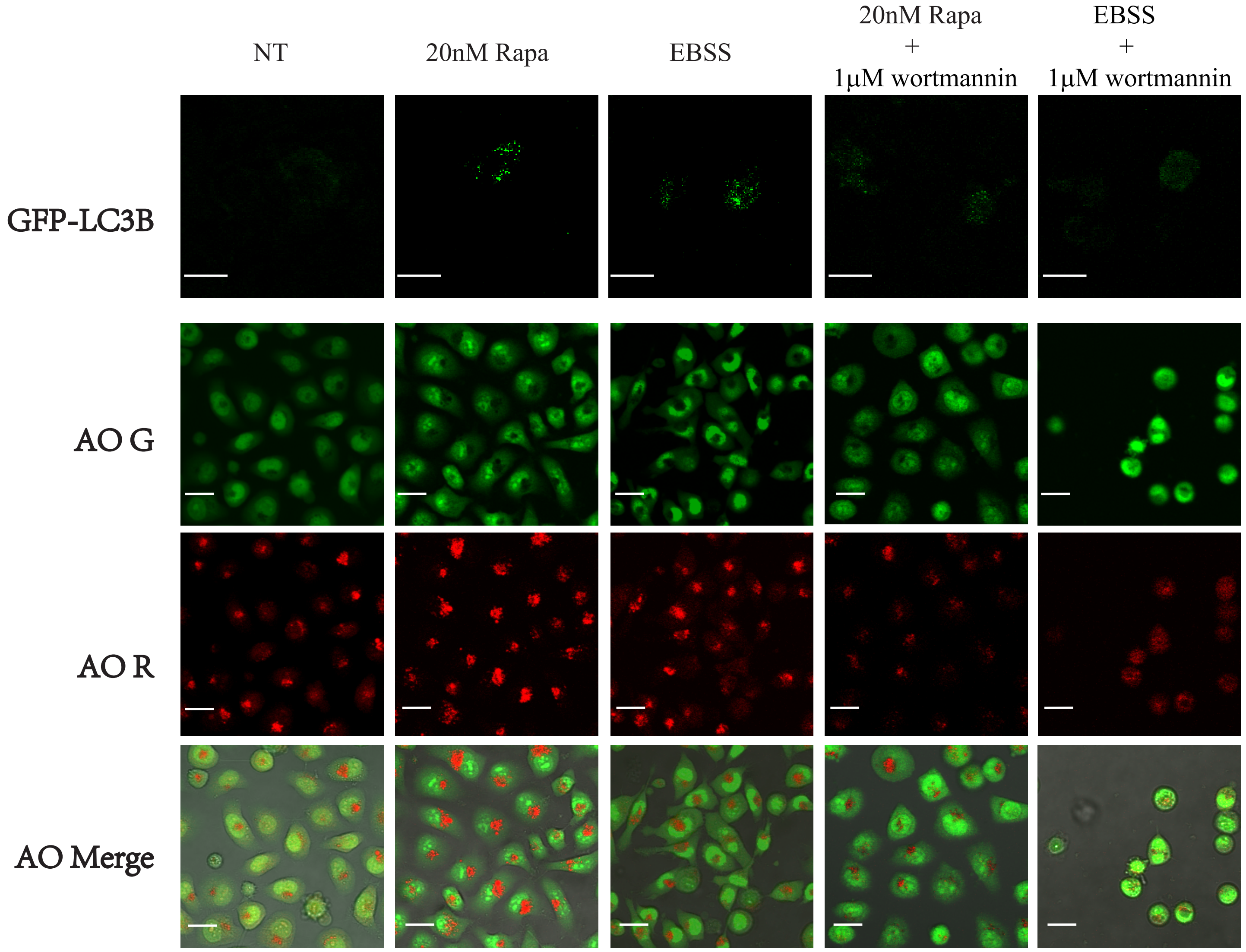

Bars=20μm

Figure S3 Beclin-1 protein in human keratinocytes

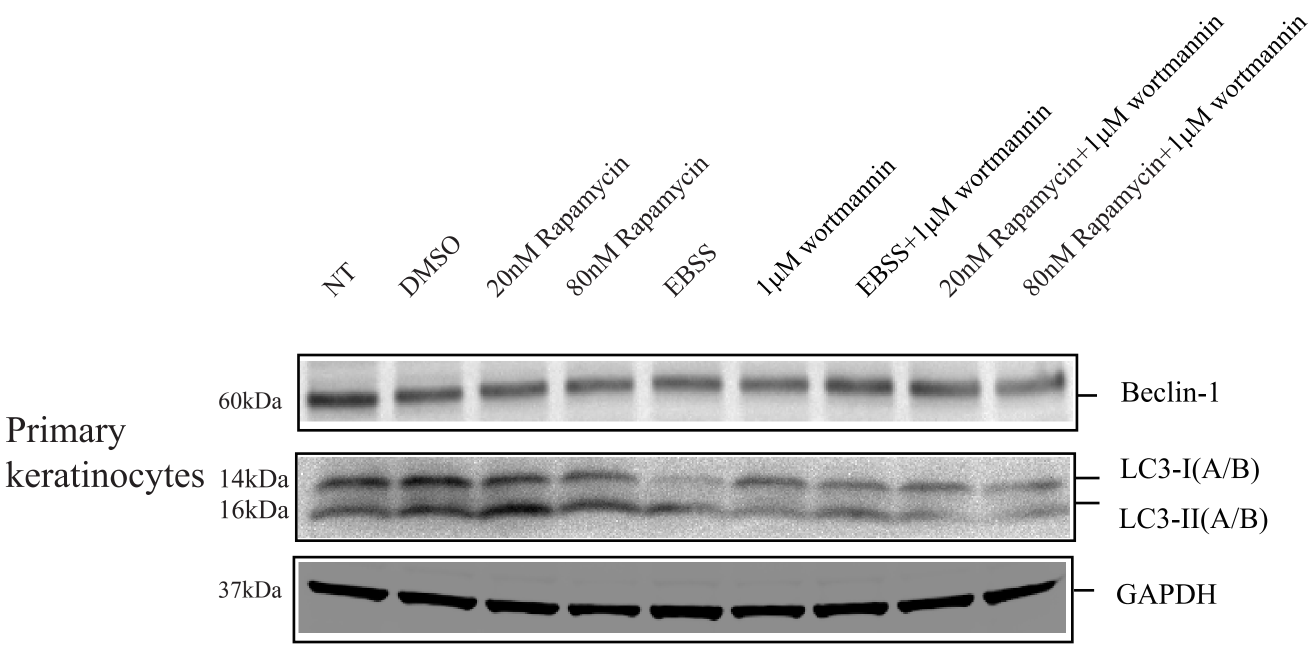

Supplement: Supplementary Information [file srep28423-s1.pdf]
